# 

**Published:** 1903-10-15

**Authors:** 


### CONTENTS.

Some Phases of Pyorrhea Alveolaris and its Treatment, *By Geo. W. Cook, B.S., D.D.S.* 487

Diet for Pyorrhea Alveolaris, *By J. Warren Achorn, M.D.* 501

President's Address, - - *By E. A. Honey, D.D.S.* 508

A New Porcelain Crown, *By J. P. Carmichael, D.D.S.* 513

"Welshers," - - - - *By Dr. H. McManus* 517

Some Unappreciated Causes of Anemia in Childhood, *By W. C. Hollopeter, A.M., M.D.* 520

The Right to Administer Drugs in Dental Practice, - 523

Fillings as Barriers to Bacteria, - - *A. E. Webster* 525

Formaldehyde in Root-Canals,, - *By B. F. Thielen* 526

Editorial, - - - - - - - - - - 528

Announcements, - - - - - - - - - 530

Obituary, - - - - - - - - - - 531

Indents, - - - - - - - - - - 534

